# 3D morphology of nematode encapsulation in snail shells, revealed by micro-CT imaging

**DOI:** 10.1038/s41598-021-82106-6

**Published:** 2021-01-28

**Authors:** P. Falkingham, R. Rae

**Affiliations:** School of Biological and Environmental Sciences, Liverpool John Moores University, Byrom St, Liverpool, L33AF UK

**Keywords:** Microscopy, Ecology, Evolution, Physiology, Zoology, Biological techniques, Imaging, X-ray tomography

## Abstract

Many parasites and hosts are embroiled in an on-going arms race that affects the evolution of each participant. One such battle is between parasitic nematodes and terrestrial gastropods which have co-evolved for 90–130 MY. Recently, snails have been shown to encase and kill invading nematodes using their shell as a defence mechanism. However, there is remarkably little known about this process in terms of understanding where, when and how nematodes are fixed within the shell. Also there has never been any attempt to observe this process using methods other than light microscopy. Therefore, we used micro CT scanning of a *Cepaea nemoralis* shell (a common host for nematodes) to 3D visualise encased nematode parasites and quantify morphological parameters. By taking this approach future studies could use micro CT scanning of fossil shells in conchology collections to understand nematode/snail co-evolution.

## Introduction

**Figure 1:**
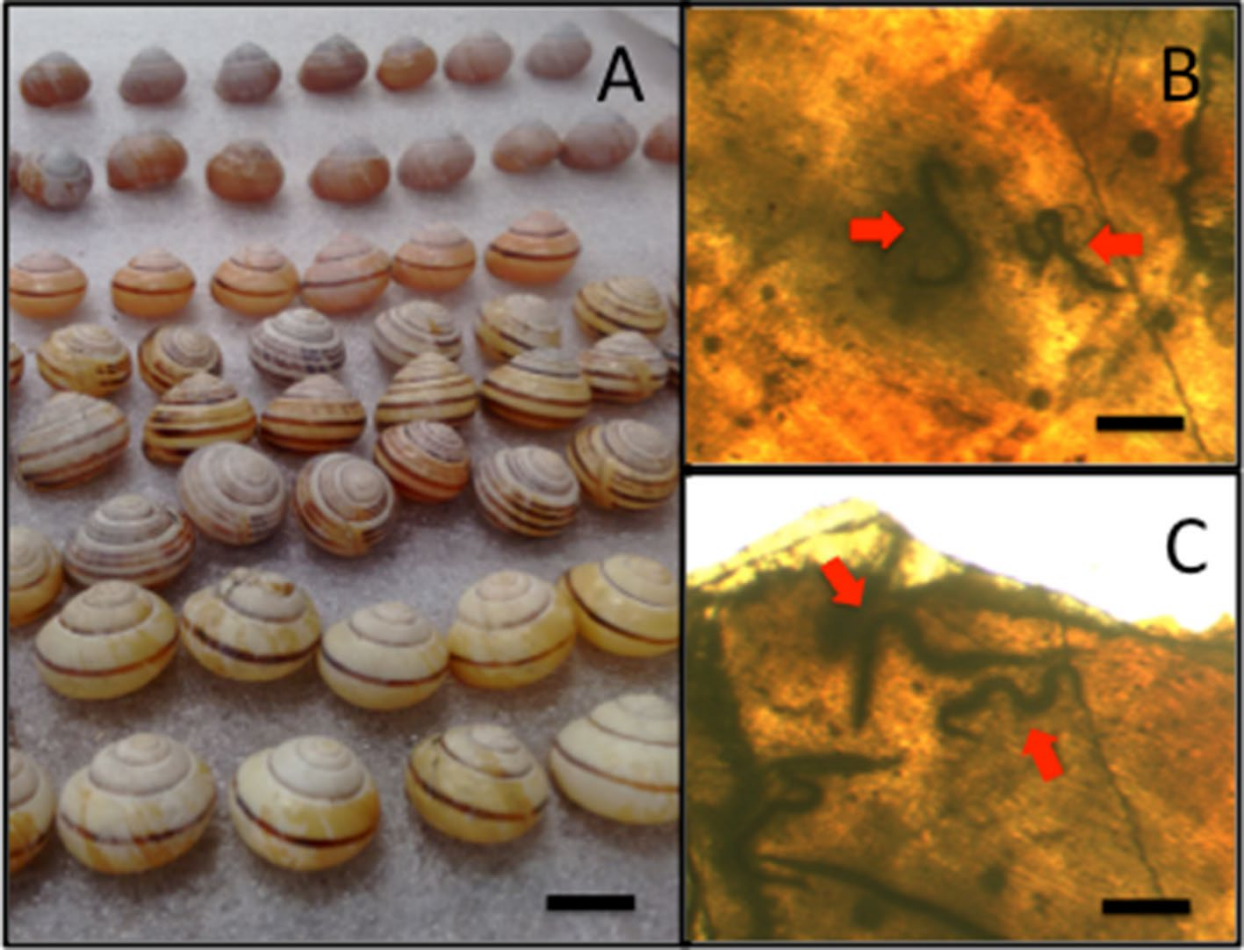
(**A**) Snails, such as *C. nemoralis*, regularly encase and kill nematodes in the inner whorl of their shell. The nematodes can be seen by using light microscopy and are fused to the inner shell (**B**,**C**). Bars represent 1 cm in (**A**) and 0.5 mm in (**B**,**C**).

The co-evolutionary arms race between host and parasite has resulted in rapid changes in the evolution of the immune system^1^. Terrestrial gastropods (slugs and snails) are parasitised regularly by flies, protozoa, trematodes and viruses^2^, but nematodes are the most prolific parasites with 108 species (representing four out of five clades of the Nematoda) using terrestrial gastropods as definitive, intermediate and paratenic hosts^3–5^. This arms race has been on going for 90–130 MY^6^. Examples include *Caenorhabditis elegans* Maupas which is thought to use slugs and snails for transport^7^ and *Angiostrongylus vasorum* Baillet (the casual agent of cardio/pulmonary disease in dogs) uses snails as intermediate hosts to facilitate transmission to mammals^8^. In order to combat parasites, terrestrial gastropods use Reactive Oxygen Species (ROS), antimicrobial peptides and lectins to kill invading parasites^9^, but in general, their immune system is poorly researched^10^. Interestingly, recent studies examining the susceptibility of snails to the commercially available biological control agent nematode parasite *Phasmarhabditis hermaphrodita* Schneider (sold as Nemaslug)^11^, observed nematodes being trapped, encased and killed by unknown cells fusing the animals to the inner part of the shell en masse^12–16^. The shell is made of an outer proteinaceous periostracum of conchiolin and crystalline calcium carbonate sub-layers^17^ and is used for shelter from extreme environmental conditions but this recent research posits the shell has been co-opted to kill nematodes^14^. Upon nematode infection, cells on the shell surface aggregate and adhere to the nematode cuticle and fuse it to the inner shell, often hundreds at a time. This was initially observed in infection experiments with the giant African snail (*Lissachatina fulica*) Férussac^12^ and has subsequently been observed in live *Cepaea nemoralis* L.^13^, *Arianta arbustorum* L.^15^ and in museum collections of *Cornu aspersum* Müller^16^ and across many representatives of the Stylommatophora^14^, even in the vestigial shell of slugs^18^. By examining shells in conchology collections nematodes over 500 years old have been observed^14^. This in vivo fossilisation process could allow an unprecedented insight into spatial and temporal changes in co-evolutionary dynamics between nematodes and snails. Also as nematode DNA can be extracted from preserved shells to aid identification to species^14,16^ the molecular evolution of nematodes could be tracked over time. As nematodes are soft bodied and do not fossilise^19,20^ this approach has huge potential however, the basic processes involved in encapsulation used to kill nematodes are poorly understood. This is primarily as the inner aperture and whorl of a snail’s shell is difficult to observe. Light microscopy has been used to view nematodes fixed and fused in shells (Fig. 1) but there have been no other techniques used to investigate this further. Hence, new, non-destructive approaches are needed. One such approach is micro computed tomography (micro CT scanning) that has been successfully used understand the structure of arachnids^21^ and ammonites^22^. Thus, we had two main aims; first, to use micro CT scanning to discover if nematodes can be viewed in the snails’ shell and second, whether any morphometric data can be gleaned from such an approach.

## Materials and methods

### Observation and scanning of nematodes encased in snail shells

A collection of approximately 1–2 year old *C. nemoralis* shells collected from sand dunes in Formby, Sefton (n = 50) (Grid Reference: SD273075) were examined for nematodes encased in the inner lip and whorl of the shells using light microscopy following standard procedures^12–16^. One *C. nemoralis* shell had a prominent nematode fixed opposite the inner whorl of the shell and was used for subsequent studies. The specimens were scanned using a SkyScan 1272, at voxel size of 19.82 µm^3^. Voltage and Current were set to 50 kV and 200 µA respectively. CT data were analysed using Dragonfly software, version 4 for Windows (http://www.theobjects.com/dragonfly/, Object Research Systems (ORS) Inc, Montreal, Canada). Thresholding was used to isolate shell material in 3D and 2D views, and the encapsulated nematode located. 3D views of the encapsulated nematode were rendered with a “hard gradient” to illustrate the morphology of the feature. 2D slices detailing the internal morphology of the encapsulation were produced with a rainbow colour map to indicate density (warmer colours indicate higher density). Tomographic data are provided in supplemental data.

## Results

**Figure 2:**
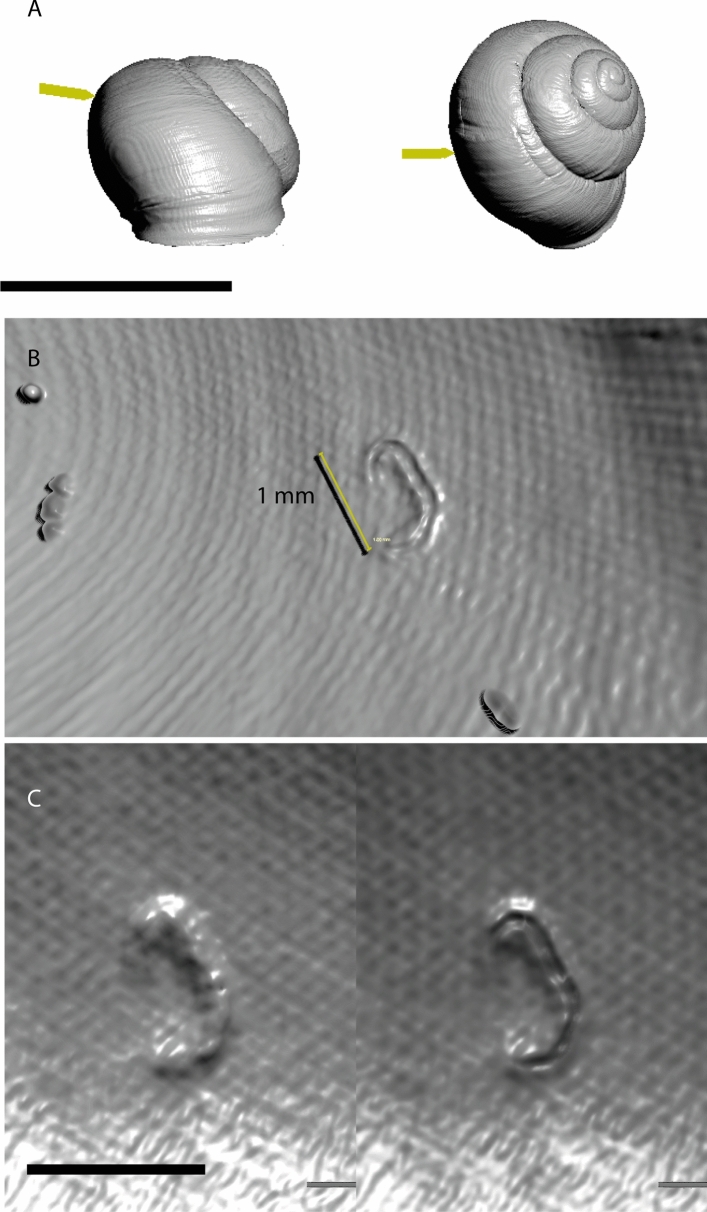
3D views of the nematode encapsulation. (**A**) Arrows indicate location of the encapsulation in anterior and dorsal views of the shell. (**B**) The encapsulation inside the shell. Fine sand grains are also adhered to the inside of the shell and are visible. (**C**) 3D view of the encapsulation at two slightly different thresholds—on the left, a broader threshold window showing the complete nature of the encapsulation, and right, a narrower thresholding window exposing the internal geometry of the encapsulation. Scale bar = 20 mm in (**A**), and 1 mm in (**B**,**C**).

**Figure 3:**
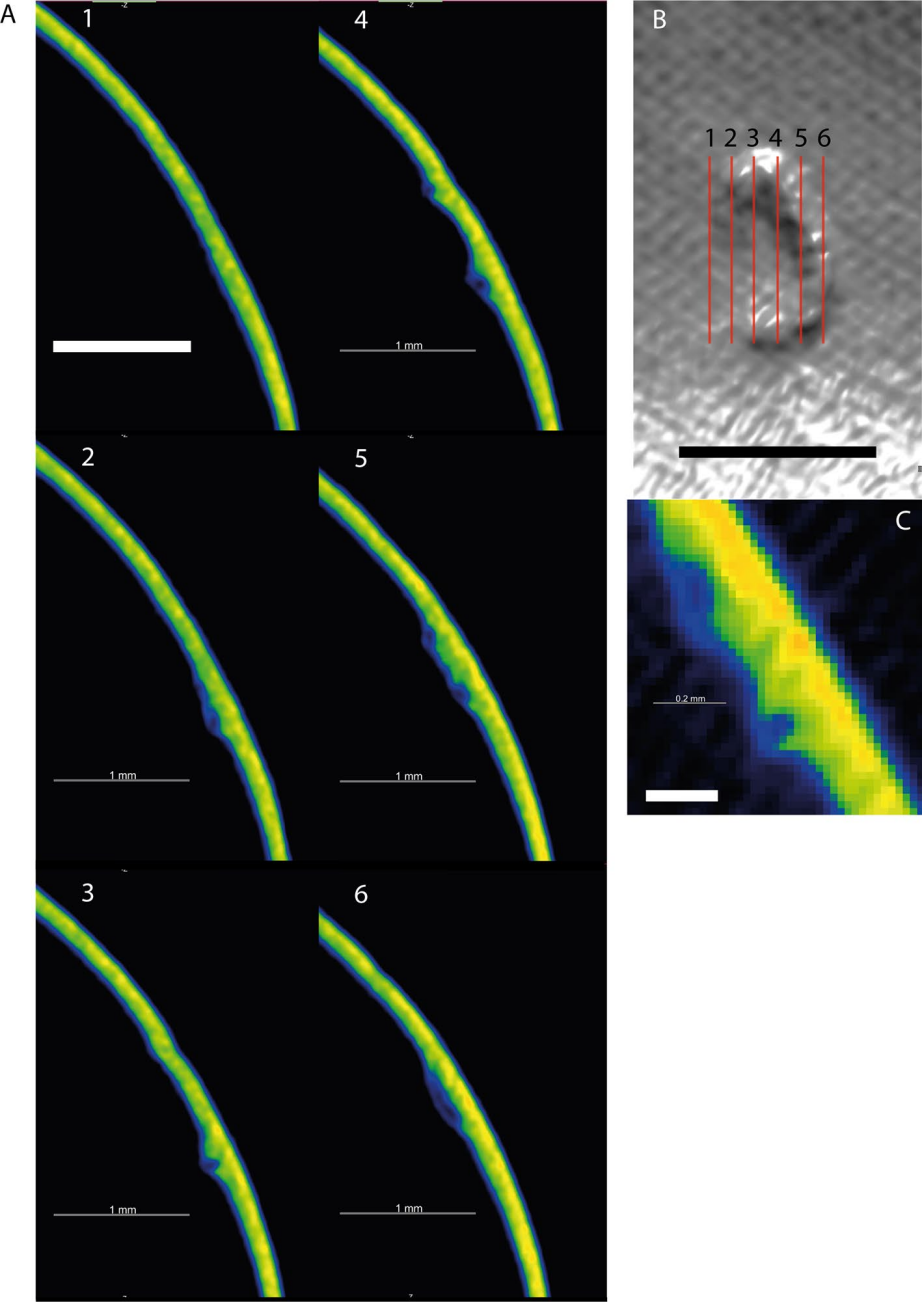
(**A**) Six sequential slices through the encapsulation and surrounding shell. The complete encapsulation, and tubular nature of the structure can be seen in slices 2–6. Slices are coloured according to density—warmer colours indicate higher density. Slice locations are shown in the upper right (**B**). (**C**) Close up slice shown as raw pixel data (non-interpolated). Scale bar in A + B = 1 mm, scale bar in C = 0.2 mm.

Prior to scanning, one *C. nemoralis* shell was identified through light microscopy as exhibiting encapsulation of an individual nematode located inside the dorsal portion of the shell (Fig. 2a; Supplementary Video S1). The feature is C-shaped, curving and tapering at both ends. The encapsulation is ~ 1 mm long and 0.2 mm in width, (Fig. 2b,c) and is raised 0.1 mm above the surrounding shell surface (Fig. 3). The outermost layer of the encapsulation is extremely thin, only a few voxels in width. The interior of the encapsulation is approximately 80 µm in width, and has higher density than the surrounding air, but lower density than the shell around it. It is clear there is a cavity produced when the nematode is covered in unknown cells (Fig. 3c). However, the lack of resolution here makes it difficult to draw firm conclusions regarding the nature of the interior of the encapsulation (see Fig. 3c).

## Discussion

The number of shells found with nematodes present was surprisingly low in our study. This is unusual. The number of shells positive for nematode encapsulation as well as the number of nematodes found per shell has been found to be high in field based studies. For example, from *C. nemoralis* collected from Merseyside, 4–60% of shells had nematodes present ranging from 1 to 152 nematodes per shell^14^. Similarly, 2–25% of *C. hortensis* shells from north Scotland had from 1 to 51 nematodes present^14^. This high infection load is not restricted to snails from the genus *Cepaea*. All shells of *C. aspersum* (n = 136) from an escargot farm in northern Ireland had nematodes present in their shells with a mean of 31 ± 2 nematodes per shell^14,16^. Snail shells hundreds of years old housed in conchology collections have nematodes encased in their shells. For example, *A. arbustorum* from 1908^15^, *C. aspersum* and *Helix pomatia* L. from 1901 and 1904^16^ respectively, as well as *C. nemoralis* from 1864 and even over 500 years old^14^ all had nematodes encased in their shells. Therefore, as nematode encapsulation is common in many members of the Stylommatophora^14^ there is ample opportunity for studying the spatial and temporal changes in nematode infection in many different species and locations using light microscopy and μCT scanning.

Previous attempts using standard light microscopy have been able to quantify nematode numbers fixed in snail shells^12–16^ but 3D visualisation and measurements of individual animals have not been possible. Using micro CT scanning we have been able to remedy this problem. The 1 mm long nematode fused to the inner shell of *C. nemoralis* was covered with a thin layer of unknown snail cells leaving a clear cavity where the nematode degrades. It was previously unknown whether snail cells would fill this void or if layers were produced on top of the lesion during the encapsulation process. This is an interesting and important discovery for future research, as this cavity will protect nematode DNA from agents responsible for degradation e.g. extreme temperatures and water^23^. Molecular analyses of museum collections have yielded fascinating insights into the evolution of many organism including humans^24^, plants^25^ and even bacterial pathogens^26^. Perhaps this system could be no different. If shells are stored correctly in conchology collections this encapsulation process could allow molecular analysis of nematodes over time using fossilised shells. In general, molecular approaches of preserved nematodes have been restricted to genotyping of helminth eggs from coprolites and archaeological digs hundreds even thousands of years old. For example, *Ascaris* eggs were extracted from coprolites from the Middle-Ages in Belgium^27^ and *Ascaris* sp. and *Trichuris* sp. eggs have been identified from environmental samples from Viking age sediment (dated 1018–1030 AD)^28^. This is due to helminth eggs being resistant to environmental stressors. From our understanding there have been no molecular analyses (other than a few genes for genotyping) of preserved nematodes at any other developmental stage as they do not fossilise. In contrast, adult stage nematodes encased in *C. nemoralis* shells over 500 years old have been observed^14^. Examination of older shells is possible and is highly likely to yield positive shells with evidence of nematode parasitism. One such group of snails to focus on could be edible land snails (e.g. *C. aspersum*), reared by humans in the late Pleistocene and Holocene and are often abundant in archeological deposits and hence museums^29^. Analysis of these shells could potentially tell us about the evolutionary history of nematodes infecting humans. For example, a common parasite of snails is *Angiostrongylus cantonensis* Chen the causal agent of human eosinophilic meningoencephalitis worldwide^30^.

Although our study using micro CT scanning was successful in providing information about nematode parasitism of snail shells there are a wealth of techniques to use in future studies including using higher resolution scanners or microscopy including Transmission Election Microscopy (TEM). Scanning Electron Microscopy (SEM) is another possibility though this would involve breaking shells to reveal the nematodes inside.

Providing these 3D observations opens up the opportunity of examining the fossil record for nematode-snail relationships, and exploring the evolution of this defence mechanism. The three dimensional nature of encapsulations like this makes it a very real possibility encapsulated nematodes might be observed in fossil snail specimens via µCT imaging. Armed with this search image, fossil snail collections stretching back hundreds of millions of years may hold important information on when this capability evolved, and how it might be spread across the snail phylogeny.

## Supplementary Information

Supplementary Information 1.

Supplementary Information 2.
