# Supplementary material for: 3D morphology of nematode encapsulation in snail shells, revealed by micro-CT imaging
Source: Sci Rep. 2021 Jan 28;11:2523. doi: 10.1038/s41598-021-82106-6 (PMC7843631; doi:10.1038/s41598-021-82106-6)
Supplement: Supplementary file 2 — Supplementary Information 2. [file 41598_2021_82106_MOESM2_ESM.pdf]

3D morphology of nematode encapsulation in snail shells, revealed by micro-CT imaging

Falkingham, P. and Rae, R.

Liverpool John Moores University, School of Biological and Environmental Sciences, Byrom St., Liverpool, L33AF, U.K.

Supplementary Info

Supplementary Video S1: Video of *C. nemoralis* shell made using micro CT scanning analysis showing a single nematode encased inside the inner whorl.
